# IUC26672-88 Treatment beyond progression in primary refractory mRCC during 1L systemic therapy (Meet-Uro 33)

**DOI:** 10.1093/oncolo/oyag286.021

**Published:** 2026-08-21

**Authors:** D Aspden, D Bimbatti, A Dalla Volta, G Roviello, R Iacovelli, G Bozza, M G Vitale, E Fantinel, A Guida, F M Deppieri, C Nasso, S Scagliarini, E Cocorocchio, B Maiorano, C Caserta, A Cavo, F Paolieri, F La Russa, L Fratino, S E Rebuzzi

**Affiliations:** Medicine and Surgery Department, University of Turin, Turin - Italy; Oncology 1 Unit, Istituto Oncologico Veneto IOV IRCCS, Padua - Italy; Unit of Medical Oncology, Department of Medical and Surgical Specialties, Radiological Sciences, and Public Health, ASST Spedali Civili di Brescia, University of Brescia, Brescia - Italy; Unit of Medical Oncology, Department of Medical and Surgical Specialties, Radiological Sciences, and Public Department of Health Sciences, University of Firenze, Florence - Italy; Università Cattolica del Sacro Cuore, Facoltà di Medicina e Chirurgia, Rome, Italy; Medical Oncology, Fondazione Policlinico Universitario Agostino Gemelli IRCCS, Rome - Italy; Medical Oncology IRCSS CROB, Rionero in Vulture, Italy - Italy; Medical Oncology Unit, Department of Oncology and Hemathology, University Hospital of Modena, Modena - Italy; Department of Oncology, Azienda Ospedaliera Universitaria Integrata di Verona, University of Verona, Verona - Italy; Medical Oncology Unit, Santa Maria Hospital, Terni - Italy; Medical Oncology Unit, AULSS 3 Serenissima, Mestre-Venice - Italy; Medical Oncology, Ospedale Santa Corona, Pietra Ligure - Italy; Deparment of Medical Oncology, AORN “A. Cardarelli”, Naples - Italy; Division of Medical Oncology, Humanitas Gavazzeni, Bergamo - Italy; Department of Medical Oncology, IRRCS San Raffaele Hospital, Milan - Italy; Oncology Unit, Santa Maria della Misericordia Hospital, Perugia - Italy; Oncology Unit, ASL3- Villa Scassi Hospital, Genoa - Italy; Department of Oncology, Hospital of Prato, Azienda USL Toscana Centro, Prato - Italy; Department of Oncology, Ospedale San Bortolo, Vicenza - Italy; Department of Medical Oncology, Centro di Riferimento, Oncologico di Aviano CRO-IRCCS, Aviano - Italy; Medical Oncology 2 Unit, Azienda Ospedaliero-Universitaria Città della Salute e della Scienza di Torino, Turin - Italy

## Abstract

**Background:**

The optimal management of metastatic renal cell carcinoma (mRCC) patients (pts) experiencing progressive disease (PD) at the 1^st^ radiological assessment during 1^st^ line therapy (primary refractory pts) remains undefined. While treatment beyond progression (TBP) may be appropriate for selected pts with clinical benefit, evidence on pts selection and survival outcomes in this setting is limited.

**Methods:**

Meet-Uro 33 is an ongoing Italian multicenter retrospective/prospective observational study of mRCC pts receiving 1^st^ line ICI-ICI, ICI-TKI or TKI. Among primary refractory pts, we compared clinical characteristics, overall survival (OS) and 2^nd^ line therapy between pts receiving TBP and those who did not (non-TBP).

**Results:**

Of 2027 pts enrolled, 242 (12%) were primary refractory: 46 (19%) TBP pts and 196 (81%) non-TBP pts. TBP pts were more likely to have IMDC favorable-risk (26% *vs* 5%) and Meet-URO score group 1 (23% vs 4%) (both: *p* < 0.001), a lower metastatic burden (41% *vs* 27% for 1 metastatic site, *p*  =  0.04) and more frequently received nivo+cabo or pembro+lenva (28% *vs* 12%, 11% *vs* 7%; *p*  =  0.02) compared to non-TBP pts. In TBP pts, the subsequent best response was PD in 56.5%, while disease control (response or stable disease) was achieved in 17.4% pts. The mOS of all primary refractory pts was 8.3 months: TBP pts had significantly longer OS than non-TBP pts (7.3 *vs* 14.1 months, *p*  =  0.0004). Overall, 122/242 pts (50%) received 2^nd^ line therapy, including 19/46 TBP pts (41%) and 103/196 (53%) non-TBP pts. No statistically significant difference in 2^nd^ line therapy choice (*p*  =  0.13) was observed, although 2^nd^ line TKI use was numerically higher in non-TBP (92% *vs* 79%).

**Conclusions:**

Among primary refractory pts, TBP was preferentially adopted in pts with favorable prognosis, low tumor burden and in those receiving highly active ICI-based combinations. TBP was associated with longer OS, despite a lower rate of subsequent 2^nd^ line therapy. These findings support the potential role of TBP in carefully selected patients with ongoing clinical benefit despite early radiological progression. Future studies are warranted to define optimal selection criteria and validate the survival impact of this strategy.

